# New Predictive Equations for Resting Energy Expenditure in Normal to Overweight and Obese Population

**DOI:** 10.1155/2019/5727496

**Published:** 2019-12-18

**Authors:** Ali M. Almajwal, Mahmoud M. A. Abulmeaty

**Affiliations:** ^1^Clinical Nutrition Program, Community Health Sciences Department, King Saud University, Riyadh, Saudi Arabia; ^2^Obesity Research and Management Unit, Medical Physiology Department, Faculty of Medicine, Zagazig University, Zagazig, Egypt

## Abstract

**Background and Aims:**

The unique demographic and dietary characteristics of modern Arabic population require development of a new predictive equation for the estimation of resting energy expenditure (REE). This study presented new equations characteristic to Saudi population.

**Methods:**

A set of predictive equations for REE was derived for 427 healthy male and female subjects (aged 18–57 ± 14 years). REE was measured (REEm) by indirect calorimetry (IC) and predicted (REEp) using nine equations. REEp was compared with REEm to determine the predictive accuracy of these equations. Using IC and anthropometrics for stepwise linear regression analysis, a new set of equations to predict REE of men and women was developed. Accuracy of the new main equations was further tested in an external sample of 48 subjects (men = 50%).

**Results:**

Using a number of parameters (bias, underprediction, overprediction, and % accurate prediction), our results suggested that almost all (9/9 in men and 7/9 in women) equations either underpredicted or overpredicted (2/9) REE. None of the already existing equations showed an acceptable REEp/REEm difference as low as 5% and an accurate prediction (∼55%) at the individual level. Based on these findings, a new prediction equation (hereafter referred to as the Almajwal–Abulmeaty (AA) equation) was developed using this study's data, after a rigorous stepwise regression analysis using the following formula: REE = 3832.955 + AdjWt (kg) × 48.037 − Ht (cm) × 30.642 + gender × 141.268 − age (years) × 4.525 [AdjWt is Adjusted body weight = (Wt − IBW)/4 + IBW. IBW is Ideal body weight; for men IBW = (Ht(cm) − 152.4) × 1.0714) + 45.36 and for women IBW = (Ht(cm)−152.4) × 0.8928) + 45.36]. The regression model accounted for approximately 70% of the variance in REEm (*R*^2^ = 0.702).

**Conclusion:**

Previous equations likely over- or underpredicted REE. Therefore, the new predictive AA equations developed in this study are recommended for the estimation of REE in young to middle-aged Saudi men and women with different body mass indexes. Future research is also required for further clinical and cross-validation of these new equations.

## 1. Introduction

Resting energy expenditure (REE) is the main component of total energy expenditure, taking into consideration the amount of energy the living body requires to maintain its dynamic functions [1]. Indirect calorimetry (IC), a noninvasive method based on the volumes of O_2_ consumption and CO_2_ production, is the gold standard for the measurement of REE. However, due to high cost of equipment and operation involved, certain equations have been usually in use to estimate energy expenditure [2–6].

Numerous equations for the prediction of REE have been recommended for general use [7], including the extensively used Harris–Benedict [2] equation, such as Owen et al. [8], Mifflin et al. [9], Bernstein et al. [10], World Health Organization (WHO) [11], Müller et al. [3], and Schofield et al. [12]. However, whether these formulae appropriately calculate REE in subjects living in affluent and modern societies and whether these are valid in population other than those originally investigated in these studies remains unclear. Evidently, major factors contributed to the individual differences of REE, such as gender, age, body composition, body size, ethnicity, physical fitness, and hormonal status, and a range of related environmental and genetic factors [13, 14]. In fact, these prediction formulae have been reported to either over- or underpredict REE in diverse population groups [15].

In addition, the abovementioned predictive equations are considered unsuitable for predicting REE in certain populations with different body mass indexes [15, 16]. These limitations are partly due to heterogeneity of the reference populations, methodological drawbacks, and also variability of REE [3]. As an example, a recent heterogeneous database of 574 energy expenditure measurements demonstrated an SD of approximately 20% of the mean values obtained in different genders and age groups [17]. The interindividual coefficient of variation of REE has been reported to be approximately 8–13% [18], which may lead to a considerably higher number of over- and underestimations of REE using prediction formulae. While Schofield's analysis has been shown to be have played a significant role in reestablishing the importance of using BMR to predict human energy requirements, and some recent, e.g., Müller et al. [3], and old, e.g., Arciero et al. [19], studies have subsequently questioned the universal use and validity of these equations. In addition, other authors [20, 21] have questioned the continued use of equations in the contemporary populations because of the secular variations in their body weight and body composition.

It is worth mentioning that none of the formulae developed to date have taken into account these population differences. Based on studies that report the possible effects of race/ethnicity [22, 23] and genetic influences [13, 14] on REE, prediction equations developed from a primarily American-European sample might not be fitting for Saudis and may over- or underestimate their energy needs [24, 25]. Consequently, we were of the view that equations that could accurately predict Saudi's REE should be developed. Based on our literature review, REE measurement has not yet been conducted in Saudi Arabia using either IC or predictive equations. This scarcity of data on REE may be due to high costs and sophisticated skills required for IC [3–6] and the absence of Saudi-specific predictive equation to calculate REE. The only studies that have been recently conducted in Saudi Arabia investigated REE in patients [24, 26], female subjects only [25], or male subjects only [27]. In addition, none of these studies focused on developing a new predictive equation specifically for Saudis. Nevertheless, most of them recommended development of a new equation because their results indicated that the Harris–Benedict equation significantly overestimated REE, while the Owen equation significantly underestimated REE, confirming previous reports published by other investigators [25]. Therefore, this study was conducted in an effort to develop predictive equations to calculate REE of normal weight and overweight/obese Saudi population.

## 2. Subjects and Methods

### 2.1. Participants and Recruitment

The study subjects were recruited from both male and female sections at the Clinical Nutrition Program, Department of Community Health Science (CHS), Main Plaza of the King Saud University, and the Medical City. Participants (aged 18–57 years) were evaluated between December 2015 and June 2017 in a cross-sectional basis. Subjects with diabetes, thyroid disorders, or any other disease that could possibly affect REE were excluded. An additional inclusion requirement was reportedly good health defined as <1 sick day/month for the past year and no major existing psychological problems or any other illness [9]. Recruitment was based on the demographic characteristics of the Saudi population, i.e., normal weight, overweight, and obesity (i.e., almost 1 : 1 : 1 and as well as 1 : 1 ratio of men and women). In addition, the age range of the sample population (18–57 years) represents the age structure of the Saudi population, i.e., aged 15–64 years represent 72% of the total population (Saudi demographic survey 2016). Ethics Committee of the College of Applied Medical Sciences (CAMS), King Saud University (KSU), granted ethical approval for this study (Reference no. 11–MED1966–02).

### 2.2. Procedures

Participants attended an orientation session before the start of the study. All participants completed consent forms and demographics and health-related questionnaires. The participants were evaluated for REE using IC, body composition using bioelectrical impedance analysis (BIA), and anthropometrics to design the REE prediction equation. REE was also estimated using the Harris–Benedict, Ireton–Jones, Carrasco, Mifflin St. Jeor, Kleiber, and Owen equations. Since we were primarily interested in predicting REE across a wide range of body weight, equations developed from samples of exclusively overweight men and women were excluded. Although additional formulae that predict REE based on body composition (e.g., fat-free mass) have previously been developed, the current study was restricted to equations consisting of readily available body measures (e.g., age, weight, and height), as these are clinically more useful and easy to perform.

### 2.3. Measures

#### 2.3.1. Self-Reported Measures

Demographics, smoking status, and reproductive health history were assessed via self-report [28]. For the purpose of strict compliance, an individual was considered Saudi if she/he was born in Saudi Arabia and reported she/he had at least three grandparents of Saudi heritage.

#### 2.3.2. Anthropometry

Details of anthropometric measurements can be found elsewhere [29]. In brief, a certified physician and a trained dietitian conducted the anthropometric measurements following standard procedures. Height was measured, while the subject stood with legs straight, feet together, shoulders relaxed, arms at sides, and head in the Frankfort horizontal plane, with buttocks, shoulder blades, and heels, and occiput resting against a vertical wall, measured (in cm) using a Seca Model 206 wall stadiometer (Seca Co, Germany). Weight was evaluated using a Seca scale with gradations of 0.1 kg. Ideal body weight (IBW) was calculated based on the following equations: for men IBW male = (Ht(cm) − 152.4) × 1.0714) + 45.36 and IBW female = (Ht(cm) − 152.4) × 0.8928) + 45.36, while Adjusted body weight (AdjWt) = IBW + (Wt − IBW)/4.

#### 2.3.3. Body Composition

Fat mass (FM) and fat-free mass (FFM) were obtained using TANITA BC-418 analyzer (Tanita Corporation, Japan). As per the manufacturers' specifications, the machine emits an electric current with 50 and 500 kHz in frequency. This multifrequency bioelectric impedance analysis measures components of body impedance, reactance, and resistance that are used to accurately calculate body water, FM, and FFM. Fat mass index (FMI) was calculated by dividing FM (kg) by height (m^2^); FFM index was calculated in the same manner (FFMI = FFM (kg)/Height (m^2^)) [29].

#### 2.3.4. REE Measurement

To determine REE, IC was performed on all patients using the QUARK RMR (COSMED, Inc., Italy). The laboratory space was maintained at 20–25°C, ensuring that each individual was physically comfortable and thus properly positioned for measurements. All participants were asked to fast at least for 12 h and also to abstain from caffeine, nicotine, and physical activity (minimum abstention from vigorous resistance exercise for 24 h). Before the test, a 20 min rest was allocated for device warming and gas, air, and turbine calibration of Quark RMR. Practically speaking, REE is equivalent to RMR.

Energy expenditure was measured for all participants, which took 16 min for each individual after excluding the first 5 min preparation/stability time. Laboratory visits were scheduled at the same time of day (between 08 : 00 and 11 : 00 am). All the assessments were completed on an outpatient basis, with participants arriving at the laboratory on the morning of testing sessions; silence was observed during session, and subjects comfortably lied in the supine position without moving or sleeping. Sessions that failed to achieve at least 5 min of steady state (variations in the VO_2_ and VCO_2_ of ≤10%) were excluded from the analysis. At any time, it was not accepted to have the % coefficient of variation in gas volumes >10%.

#### 2.3.5. REE Calculation

REE was also calculated using equations that included body weight, height, age, sex, FFM, and/or FM. The exclusion criteria for these equations were as follows: equation using age range of <12 yrs or only elderly; those using only one sex, those having normal weight based on Cole et al. [30] (not applicable to large databases of Schofield and Harris and Benedict), those with insufficient information, those considering only a specific ethnic group (other than white), based on small sample size (*n* < 50); impractical or suspected body composition as a variable, glucose concentrations, or diabetes as a variable, total energy expenditure, athletes, and duplicate publications.

For each subject, the REE was predicted in kcal/day by using the selected equations and then compared with measured REE. The actual body weight during IC measurement was used for this calculation. Demographic and anthropometric data were used to calculate REE using predictive equations developed by Harris and Benedict [2], Harris and Benedict equation reevaluated by Roza et al. [31], Bernstein et al. [10], Owen et al. [8], Mifflin et al. [9], Schofield (weight only), Schofield (both weight and height) [12], FAO/WHO/UNU (weight only), and FAO/WHO/UNU (both weight and height) [11] (Table 1).

### 2.4. External Validation

A total of 48 subjects (men = 50%) were used for external validation of the main new equations. The validation population was recruited from visitors of therapeutic nutrition clinic at CAMS, KSU. The same inclusion criteria were applied, and the same measures were recorded as the original study population.

### 2.5. Statistical Analysis

All the data were analyzed using SPSS (version 23; SPSS Inc., Chicago, IL, USA). Continuous variables were expressed as means (SD), and dichotomous variables were expressed as percentages and frequencies. The accuracy of predictive formulae at the individual level was defined as percentage of the subjects whose predicted RMR was within ±10% of the measured RMR [32, 33]. The degree of agreement between the measured and predicted REE was evaluated using Bland–Altman limits during the agreement analyses. Limits of agreement were defined as the mean difference ±2.0 SD. The estimated accuracy was defined as “the percentage of the subjects whose REEp was within ±10% of REEm.” Under- and overestimation were defined as <10% and >10% of REEm, respectively [34]. Predictive equations were developed and compared with commonly used equations for validation [9, 33]. The method suggested by Bland and Altman [35] was used to plot the agreement between (a) IC and Harris and Benedict equation (1919 and 1984), (b) IC and Bernstein equation, (c) IC and Owen equation, (d) IC and Mifflin equation, (e) IC and Schofield equation, and (f) IC and WHO equation, in addition to IC and the main two new equations (AA_1 and AA_FFM). The Bland–Altman method gives calculation for mean difference between two measurement methods (the bias) and 95% limits of agreement as the mean difference (±1.96 SD). The values are expressed as absolute (kcal) and percentage (%). We used multiple stepwise regression analysis to derive the new prediction equation (hereafter designated as “Almajwal–Abulmeaty” (AA equation)) to estimate REE based on age, gender, weight, and height as independent variables [9, 32–34]. We calculated Pearson's coefficient of determination (*R*^2^) and residual standard deviation (RD) as goodness-of-fit measures between the predicted and measured regression equations used.

## 3. Results

### 3.1. Baseline Characteristics of the Participants

**Figure 1:**
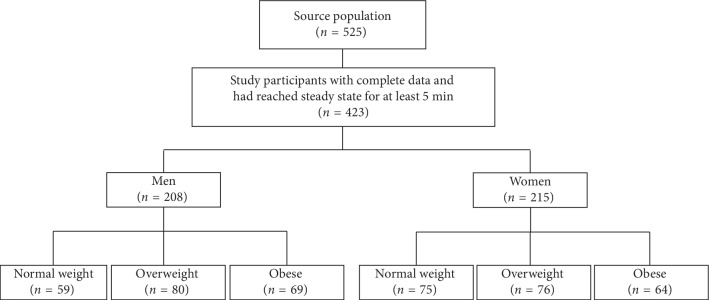
STROBE flowchart of the study participants.

Among 510 participants, the inclusion criteria were eligible for 423 participants (men 49%) (Figure 1).  Table 2 shows the characteristics of the 423 Saudi men and women, with a mean BMI of 28.5 ± 6.1 kg/m^2^. All anthropometric and body composition parameters significantly differed between men and women and between the three BMI groups, except for height (*P*, for all trends <0.05).

### 3.2. Agreement between Measured and Predicted REE (REEm and REEp)

We evaluated differences in the following measures: bias (difference between REEp and REEm) (kcal/day), percent bias (REEm − REEp by equations multiplied 100/REEp by equations), underestimation (percentage of all subjects whose REEp was <90% of REEm), overestimation (percentage of all subjects whose REEp was >110% of the measured REEm), and accurate prediction (percentage of all subjects whose REEp was within 90% to 110% of REEm).

Table 3 shows RMR (kcal/day), bias (kcal and %), maximum values found for positive error (overprediction) and negative error (underprediction), percentage accurate predictions, and percentage under- and overpredictions. In men, REEp by all 10 predictive equations was lower than REEm, the lowest being by the Bernstein equation (491.0 kcal/day), followed by Owen (251 kcal/day), Mifflin (199 kcal/day), H-B^1919^ (92.8 kcal/day), HB^1984^ (76.8 kcal/day), Schofield (Wt, Ht) (28.2 kcal/day), Schofield (Wt) (24.7 kcal/day), WHO (Wt) (17.2 kcal/day), and WHO (Wt, Ht) (16.8 kcal/day). The number of underpredictions within ±10% range varied from 90.4% (Bernstein equation) to 20.2% (WHO^W^ equation) in men and from 82.3% (Bernstein equation) to 17.2% (each Schofield^W^ and WHO^W^ equations) in women. The number of overpredictions within ±10% range varied from 23.6% (WHO^W^ equation) to 0% (Bernstein equation) in men and from 27.4% (WHO^W^ equation) to 0.5% (Bernstein equations) in women.

The percentage of accurate prediction varied greatly for all 10 predictive equations. The equations with the highest percentage accurate predictions (>55%) were WHO^WH^, WHO^W^, Schofield^WH^, and Schofield^W^ equations, respectively, having % accurate prediction of 56.3, 56.3, 56.3, and 55.8, followed by the equations of H-B^1919^ (55.3%), H-B^1984^ (54.8%), Mifflin (44.2%), Owen (37.0%), and Bernstein (9.6%). In women, out of 10 equations, two (WHO^W^, Schofield^W^) overestimated REE (mean REEp > REEm) although the difference between the means was not significant (*P* > 0.05). The remaining eight equations underestimated REE (REEp < REEm) with a statistically significant difference (*P* for all trends, <0.05). Contrary to men, equations of H-B^1984^ for female participants had the highest accurate prediction (62.3%), followed by H-B^1919^ (61.4%), WHO^WH^ (58.6%), Schofield^WH^, (58.6%), Schofield^W^ (55.8%), Mifflin (52.6%), Owen (37.7%), and Bernstein (17.2%). All the prediction equations used in the current study significantly underestimated the measured REE (*P* rends for all < 0.001) in men (Table 3).

### 3.3. Bland–Altman Plots

**Figure 2:**
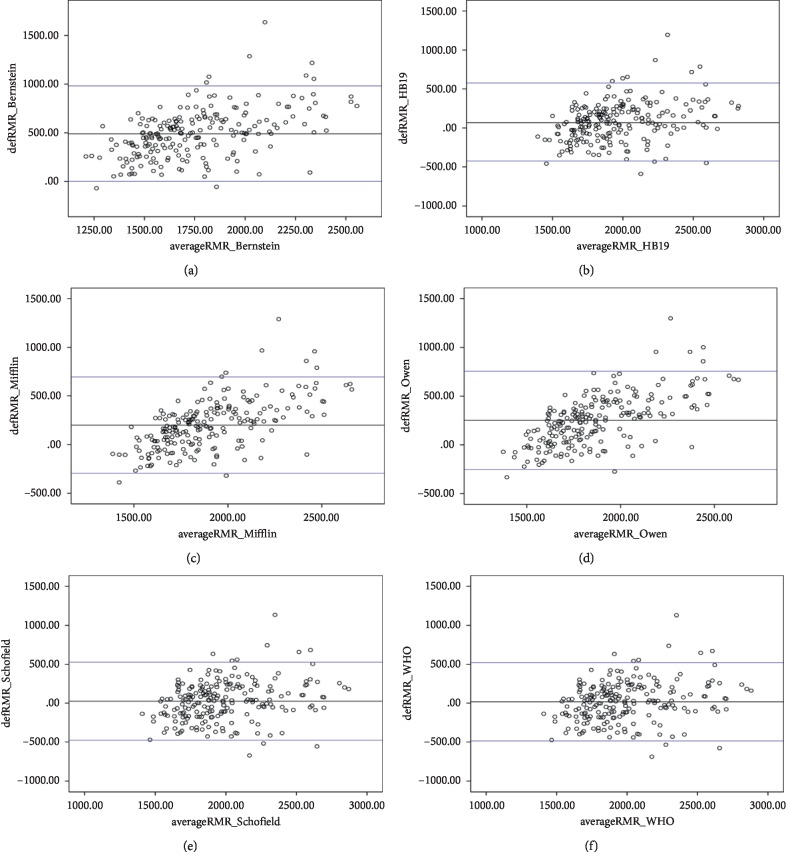
Bland–Altman plots comparing indirect calorimetry (IC) and the following prediction equations for the basal metabolic rate in Saudi men: (a) Bernstein; (b) HB; (c) Mifflin; (d) Owen; (e) Schofield (Wt only); (f) WHO (Wt only).

**Figure 3:**
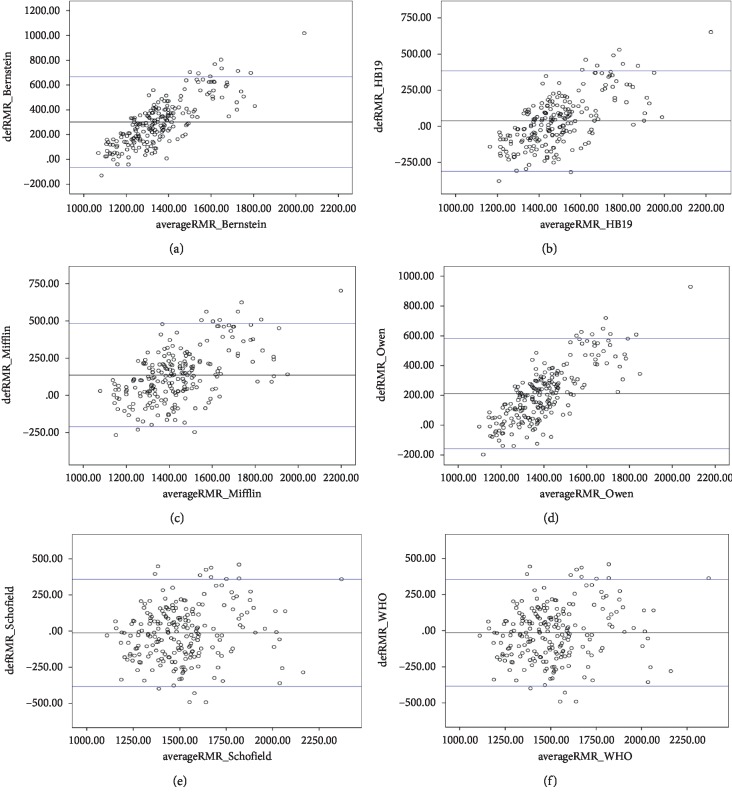
Bland–Altman plots comparing indirect calorimetry (IC) and the following prediction equations for the basal metabolic rate in Saudi women: (a) Bernstein; (b) HB; (c) Mifflin; (d) Owen; (e) Schofield (Wt only); (f) WHO (Wt only).

Figures 2 and 3 show the Bland–Altman plots comparing REE measurements obtained by IC (REEm) and by prediction equations (REEp) in men and women, respectively. REEm and REEp were significantly different. In men and women, the Bernstein equation revealed the highest underprediction (491.0 ± 250.0 kcal/day; bias of 33.5% and 301.3 ± 186.9 kcal/day; and bias of 24.7%, respectively). In contrast, the WHO (Wt, HT), WHO (Wt), Schofield (WT, HT), and Schofield (Wt) were the most accurate compared to IC in men, whereas HB (1984), HB (1919), Schofield (Wt), and Mifflin were the most accurate equations in women.

### 3.4. Development of the New REE Prediction Equations

When combining both men and women, REE significantly correlated with age (*r* = −0.230, *P*=0.01), height (*r* = 0.614, *P* < 0.001), weight (*r* = 0.730, *P* < 0.001), and gender (*r* = 0.432, *P* < 0.001).  Table 4 presents all the values of this correlation matrix. Other parameters (i.e., BMI, FFM, and FM) were also strongly correlated. However, adding any additional parameter had no significant effect on the regression model. For example, we excluded BMI from the final regression model because calculating the BMI will add another step in the clinical setting. Thus, age, height, and weight were retained in the final regression model as these are the parameters that can be easily measured in both population analysis and in clinical settings [9]. Each of the variables included in the equation significantly and independently contributed to the model (*P*, for all trends <0.01). Of no surprise, the best REE predictor was body weight (*r* = 0.730). Of the remaining variables, height demonstrated the next strongest relationship to REE (*r* = 0.614; *P* < 0.01), followed by gender (*r* = 0.432, *P* < 0.001) and age (*r* = −0.230; *P* < 0.01). The results of the multiple regression analysis produced the equations in predicting REE (kcal/day), as shown in the multivariate analysis of all study population, and the variables included in the equations significantly and independently contributed to the model (*P*=0.001). Stepwise entry of anthropometric variables revealed that adjusted weight, height, gender, and age increases *R*^2^ of this model from 0.620 to 0.702, as shown in Table 5, equation 1. Other body composition variables (TBW, FFM, and FM) that were considered in the analyses increased *R*^2^ to 0.706, but for clinical ease and slight increase in *R*^2^, the much simpler equation 1 was considered. After dividing the sample into normal weight and overweight/obese categories, another set of equations were created but *R*^2^ < 0.7 (Table 5). Separate use of stepwise multiple regression analysis for male or female subjects created equations with *R*^2^ < 0.54 (Tables 6 and 7).

### 3.5. Validation of the New Equations

**Figure 4:**
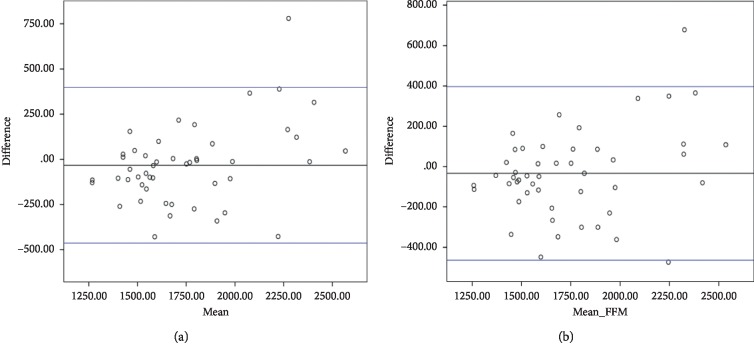
Bland–Altman plots showing the agreement of indirect calorimetry (IC) and the new equations for resting metabolic rate in the population used for external validation: (a) AA_1 and (b) AA_FFM.

Table 8 represents the general characteristics of the study population used for testing the accuracy of the new equations. As shown in Table 9, the new AA_1 and AA_FFM equations showed a lower bias and % of bias, as well as a higher accurate estimation and correlation with results of IC than Mifflin, Owen, and Bernstein equations. There was a significant difference between the means of measures by IC and estimations by Mifflin, (*P* < 0.001; 95% CI = 73.05 to 206.31), Owen (*P* < 0.001; 95% CI = 134.49 to 264.70), and Bernstein equations (*P* < 0.001; 95% CI = 303.25 to 447.64). The Bland–Altman plot (Figure 4) showed the agreement between the IC measurements and the new equations.

## 4. Discussion

This study compared the accuracy of nine predictive equations. Therefore, REEm was compared with REEp in a sample composing Saudi men and women. This study revealed that the widely used REE prediction equations used in this study cannot be used for a population living in an affluent and modern society in Saudi Arabia. In this study, we found significant and systematic over- and underpredictions between the predicted and measured REE values.

The percentage accurate predictions varied between equations, from 56.3% (for each WHO^WH^, WHO^H^, and Schofield^WH^) to 9.6% (Bernstein) in men and from 62.3% (HB^1984^) to 17.2% (Bernstein) in women. The bias for predictive equations used in this study varied from 33.5% (Bernstein) to 4.3% (H-B^1919^) in men and from 24.7% (Bernstein) to 0.4% (Schofield^W^) in women (Table 2). These results suggest no predictive equation was suitable to predict REE in men as all 10 predictive equations demonstrated bias of >1%. In women, only two equations (WHO^W^ and Schofield^W^) had bias of <1% (Table 3). Our findings (Table 3) showed that some of the predictive equations (WHO^WH^, WHO^W^, Schofield^WH^, and Schofield^W^) had relatively high % accurate prediction (>50 and <60%) but with >1% bias and can be used for REE estimation at the population levels in Saudi men. Similarly, in women, our data (Table 4) showed that some equations (H-B^1984^, H-B^1919^, WHO^WH^, WHO^W^, Schofield^WH^, Schofield^W^, and Mifflin) had relatively high percentage accurate prediction (>50 < 60%), but only two equations (WHO^W^ and Schofield^W^) had bias of <1% and can be used for REE estimation at the population levels for Saudi women. However, none of the 10 equations used for comparison in the current study can be reliably used to assess individual differences, as they are likely to provide inaccurate results in clinical settings.

Based on these data, four main lines of arguments can be drawn: (1) all nine predictive equations used in this study underpredicted REE in men, while most (eight out of nine) predictive equations underestimated REE for women, but two (WHO^W^ (Wt) and Schofield^WT^) overpredicted REE in women; (2) predictive equations that underpredicted REE in men mostly overpredicted those in women; (3) predictive equations overpredicted REE at the higher REE level (e.g., men in this study) and underpredicted REE mostly at lower REE levels (as in women), an observation in contrast to that by Müller et al. [3]; and (4) only two of these predictive equations (WHO^W^ and Schofield^W^) showed an acceptable REEp/REEm difference of <1% and an accurate prediction (∼55%) at the individual level [36]. Therefore, we would recommend the use of the equations we developed (AA equation) based on a mixture of men and women and that would offer the possibility to cover individuals with a wider BMI range (18–31 kg/m^2^).

WHO formulae are widely used to predict REE. These equations are based on considerably a large number of REE measurements performed in the twentieth century. Although some more recent formulae have been provided by other authors, one shortcoming is that none of these equations were based on a comparably large database [37]. The Owen equation was derived on 44 otherwise healthy obese and women between 18 and 65 years of age and 60 obese and lean men between 18 and 82 years of age [8]. The Mifflin equation was derived from data of 498 healthy subjects (251 men/247 women; aged 19 to 78 years, with 234 obese and 264 had normal weight) [9]. These equations are commonly used in clinical practice. The accuracy rates of the Owen equation are 37.0% and 37.7%, respectively; men and women and those of the Mifflin equation are 44.2% and 52.6%, respectively. The accuracy rates of these two equations are comparable to the recently conducted studies, e.g., Rao et al. [38], in Chinese population. However, these equations had a higher percentage bias in both Saudi men and women (Table 4) and, therefore, cannot be recommended in Saudi population and REE prediction in the clinical setting.

The WHO equation was developed on a study of young Europeans, mostly police and military recruits, with a high proportion (45%) being of the Italian descent [32]. In our study, the subjects were Saudi adults from the civil community. This might explain that despite the relatively higher accuracy of WHO^WT^ and WHO^W^ equations in women (56.8% and 55.3%, respectively), only WHO^W^ had a percentage bias of <1.0. Therefore, WHO equations (both WHO^WT^ and WHO^W^) may not be suitable for, at least, Saudi men. As also argued by De Oliveira et al. [39] and Owen et al. [8], WHO^W^ is the most suitable equation for normal weight individuals [40], and with some restrictions, Mifflin's equation has been indicated suitable for REE estimation in normal, overweight, and obese individuals in the United States [7]; however, this might not be as much true in other populations. Based on some previous studies [41–43], there are no suitable equations for overweight/obese persons [39]. Mifflin's equation was developed using data from 498 individuals classified as normal, overweight, and obese/seriously obese [9]; despite its validation in other communities, it is most suitable for the American individuals with a BMI range of 25 to 40 kg/m^2^ and aged 16–65 years [34]. However, we did not observe a good relationship between Mifflin's equation and overweight individuals in the present study. The Mifflin equation was proved as a good equation for estimating REE in western population with various body masses [7]; however, this was not the case in our population (Tables 4 and 9). The underestimation while using the Owen equation was not unexpected because of its reliability for normal weight populations, which presents lower REE than for that for obese individuals [40].

This study has a number of strengths. The sample size (427 individuals) was large enough for subgroup (men and women) analyses. Furthermore, the data were derived from otherwise healthy individuals (normal BMI, overweight, and obese), and therefore, the study population may be representative for a wider BMI subjects and hence has large generalizability. The prediction equation we developed in this study is the first, to our knowledge, that is specifically adjusted for Arab ethnicity. This study included women representing a wide range of body weight, a fact which may increase the generalizability of the newly developed equation to other samples. Finally, we carefully controlled for the effects of several potentially important confounders, such as menopausal status, menstrual cycle, pregnancy/lactation, other relevant medical conditions, and the possible thermogenic effects of food and nicotine. However, there are several limitations in this study as well. First, the investigation did not include subjects with BMI <18.5 (underweight), which is still relatively a substantial fraction of the total population and their underrepresentation may cause a decrease in the generalizability of the new equation. Second, the sample was limited to men and women aged 18–57 years; thus, the predictive value of the new equation in estimating REE among older men and women may be uncertain. Future investigations should focus on the impact of ethnicity on the accuracy of prediction equations that have been developed for older Saudi men and women, e.g., study by Arciero et al. [19]. For this future, studies should focus on examining whether equations for predicting REE should be modified/adjusted for use in other ethnic groups. In addition, although the AA equation may provide good estimates of REE needs, a nontrivial amount of REE variance remains unaccounted. Nevertheless, REE variance accounted using the prediction equation is consistent with that of the previous studies.

In conclusion, we found in the present study that REE predictive equations are only accurate in approximately half the individuals. The WHO equation is advised for use up to BMI 30 kg/m^2^, and HB^1918^ equation is advised for obese individuals (BMI of >30 kg/m^2^). Measuring REE with indirect calorimetry is the preferred option and should be used when the facility is available as well as feasible for the optimization of nutritional support in hospital in- and outpatients with different degrees of malnutrition. In case of nonavailability/unfeasibility of the facility, we recommend use of a population-specific predictive equation. Future validation of other equations developed in this study is required using adequate samples.

## Figures and Tables

**Table 1:** Published predictive equations selected and used for the current study.

Equation name	Abbreviated/rephrased in this paper	Full equation
Harris and Benedict [2]	H-B^1919^	M: WT × 13.7516 + HTCM × 5.0033 − age × 6.755 + 66.473 = kcal/d
		F: WT × 9.5634 + HTCM × 1.8496 − age × 4.6756 + 655.0955 = kcal/d

Harris and Benedict [31]	H-B^1984^	Harris and Benedict equation reevaluated by Roza et al.
		M: 13.397 × WT + 4.799 × HTCM − 5.677 × age + 88.362
		F: 9.247 × WT + 3.098 × HTCM − 4.33 × age + 477.593

Bernstein et al. [10]	Bernstein	M: 11.02 × WT + 10.23 × HTCM − 5.8 × age − 1032 = kcal/d
		F: 7.48 × WT − 0.42 × HTCM − 3 × age + 844 = kcal/d

Owen et al. [8]	Owen	M: WT × 10.2 + 879 = kcal/d
		F: WT × 7.18 + 795 = kcal/d

Mifflin et al. [9]	Mifflin	9.99 × WT + 6.25 × HTCM − 4.92 × age + 166 × gender − 161 = kcal/d

Schofield [12]	Schofield^W^	Equations using weight only
		M: 30–60 y: 0.048 × WT + 3.653 = MJ/d
		*M* ≥ 60 y: 0.049 × WT + 2.459 = MJ/d
		F: 30–60 y: 0.034 × WT + 3.538 = MJ/d
		*F* ≥ 60 y: 0.038 × WT + 2.755 = MJ/d

Schofield [12]	Schofield^WH^	Equations using weight and height
		M: 30–60 y: 0.048 × WT − 0.011 × HTM + 3.67 = MJ/d
		*M* ≥ 60 y: 0.038 × WT + 4.068 × HTM − 3.491 = MJ/d
		F: 30–60 y: 0.034 × WT + 0.006 × HTM + 3.53 = MJ/d
		*F* ≥ 60 y: 0.033 × WT + 1.917 × HTM + 0.074 = MJ/d

FAO/WHO/UNU [11]	WHO^W^	Equations using weight only
		M: 30–60 y: 11.6 × WT + 879 = kcal/d
		*M* ≥ 60 y: 13.5 × WT + 487 = kcal/d
		F: 30–60 y: 8.7 × WT + 829 = kcal/d
		*F* ≥ 60 y: 10.5 × WT + 596 = kcal/d

FAO/WHO/UNU [11]	WHO^W^	Equations using weight and height
		M: 30–60 y: 11.3 × WT − 16 × HTM + 901 = kcal/d
		*M* ≥ 60 y: 8.8 × WT + 1128 × HTM − 1071 = kcal/d
		F: 30–60 y: 8.7 × WT − 25 × HTM + 865 = kcal/d
		*F* ≥ 60 y: 9.2 × WT + 637 × HTM − 302 = kcal/d

**Table 2:** Characteristics of study population.

Variables	Total Mean ± SD (*n* = 423)	Men				Women				*P* value
		Mean ± SD (*n* = 208)				Mean ± SD (*n* = 215)				
		Normal Wt (*N* = 59)	Over Wt (*N* = 80)	Obesity (*N* = 69)	*P* value	Normal Wt (*N* = 75)	Over Wt (*N* = 76)	Obesity (*N* = 64)	*P* value	
Age (year)	28.1 ± 8.0	23.6 ± 5.6	26.4 ± 8.7	25.0 ± 6.8	NS	28.2 ± 5.3	31.5 ± 8.1	33.6 ± 81	*P* < 0.01	*P* < 0.01
Ht (cm)	164.4 ± 8.5	170.9 ± 5.9	170.2 ± 5.4	171.5 ± 6.4	NS	158.0 ± 5.5	159.0 ± 53	157.4 ± 5.6	NS	*P* < 0.01
Wt (kg)	77.4 ± 19.0	65.5 ± 6.6	79.5 ± 6.5	108.5 ± 14.6	*P* < 0.01	56.3 ± 5.8	69.4 ± 6.2	85.7 ± 12.1	*P* < 0.01	*P* < 0.01
BMI (kg/m^2^)	28.5 ± 6.1	22.4 ± 1.8	27.4 ± 1.4	36.9 ± 5.0	*P* < 0.01	22.5 ± 1.7	27.4 ± 1.4	34.6 ± 4.3	*P* < 0.01	*P* < 0.05
IBW	57.8 ± 9.3	65.4 ± 6.4	64.5 ± 5.8	66.0 ± 6.9	*P* < 0.01	50.4 ± 4.9	51.4 ± 4.7	49.9 ± 5.1	NS	*P* < 0.01
AdjWt	62.7 ± 10.1	65.4 ± 5.9	68.3 ± 5.7	76.6 ± 7.2	*P* < 0.01	51.9 ± 4.8	55.9 ± 4.9	58.9 ± 5.9	*P* < 0.01	*P* < 0.01
WC (cm)	87.8 ± 14.9	76.8 ± 7.8	89.7 ± 8.1	111.4 ± 10.5	*P* < 0.01	73.1 ± 6.3	82.4 ± 6.6	93.8 ± 8.8	*P* < 0.01	*P* < 0.01
HC (cm)	104.9 ± 13.6	92.7 ± 5.6	102.8 ± 12.7	120.4 ± 10.5	*P* < 0.01	93.8 ± 6.9	103.6 ± 6.1	116.5 ± 9.6	*P* < 0.01	NS
WHR	0.76 ± 0.1	0.83 ± 0.1	0.88 ± 0.1	0.93 ± 01	*P* < 0.01	0.78 ± 0.1	0.80 ± 0.1	0.81 ± 0.1	*P* < *0.05*	*P* < 0.01
MAC (cm)	30.4 ± 4.6	26.3 ± 2.4	28.8 ± 2.2	33.5 ± 4.4	*P* < 0.01	27.1 ± 2.4	31.1 ± 2.6	36.0 ± 4.1	*P* < 0.01	*P* < 0.05
TST (mm)	27.2 ± 8.9	17.1 ± 6.7	25.4 ± 6.6	34.3 ± 6.3	*P* < 0.01	22.3 ± 6.3	29.5 ± 6.7	34.0 ± 7.3	*P* < 0.01	*P* < 0.05
MAMA (cm^2^)	39.2 ± 13.3	35.5 ± 10.8	35.0 ± 8.0	42.5 ± 14.7	*P* < 0.01	32.9 ± 8.5	38.4 ± 8.9	52.2 ± 17.7	*P* < 0.01	*P* < 0.05
PBF (%)	31.6 ± 9.4	17.6 ± 5.1	25.1 ± 4.0	34.8 ± 5.2	*P* < 0.01	30.0 ± 4.6	37.6 ± 3.2	43.8 ± 6.0	*P* < 0.01	*P* < 0.01
FM (kg)	25.0 ± 11.3	11.6 ± 4.0	19.9 ± 3.8	38.0 ± 10.0	*P* < 0.01	17.1 ± 3.9	26.2 ± 4.0	37.3 ± 7.9	*P* < 0.01	*P* < 0.05
FMI	9.3 ± 4.3	4.0 ± 1.3	6.9 ± 1.3	13.0 ± 3.5	*P* < 0.01	6.8 ± 1.4	10.3 ± 1.3	15.0 ± 3.0	*P* < 0.01	*P* < 0.01
FFM (kg)	52.2 ± 11.5	53.4 ± 5.2	59.3 ± 5.2	70.0 ± 7.7	*P* < 0.01	39.2 ± 3.0	43.3 ± 3.3	48.4 ± 5.1	*P* < 0.01	*P* < 0.01
FFMI	19.2 ± 3.0	18.3 ± 1.2	20.4 ± 1.1	23.8 ± 2.4	*P* < 0.01	15.7 ± 0.9	17.1 ± 0.8	19.5 ± 1.7	*P* < 0.01	*P* < 0.01
TBW (L)	47.1 ± 19.5	57.0 ± 8.5	52.6 ± 5.4	49.1 ± 5.0	*P* < 0.01	40.8 ± 11.8	39.9 ± 7.6	39.3 ± 4.4	NS	*P* < 0.01
RMR (kcal/day)	1749.8 ± 391.6	1762.5 ± 224.5	1932.4 ± 293.4	2278.7 ± 344.3	*P* < 0.01	1366.4 ± 159.0	1526.7 ± 216.2	1647.6 ± 261.4	*P* < 0.01	*P* < 0.01
RQ	0.84 ± 0.1	0.79 ± 0.1	0.79 ± 0.1	0.81 ± 01	NS	0.72 ± 0.1	0.71 ± 0.1	0.72 ± 0.1	NS	*P* < 0.01
VO_2_ (ml/min)	258.2 ± 56.0	258.5 ± 33.7	283.1 ± 42.2	331.5 ± 50.0	*P* < 0.01	204.6 ± 24.1	227.1 ± 34.5	246.3 ± 39.6	*P* < 0.01	*P* < 0.01
VCO_2_ (ml/min)	196.4 ± 52.7	203.0 ± 29.1	222.9 ± 40.0	268.2 ± 47.2	*P* < 0.01	146.7 ± 19.6	163.4 ± 27.9	176.5 ± 29.5	*P* < 0.01	*P* < 0.01

Significance difference between subgroups was assessed by the one-way ANOVA test while that between men and women was tested by the independent samples *t*-test. NS, nonsignificant; Ht, height; Wt, weight; BMI, body mass index; WC, waist circumference; HC, hip circumference; WHR, waist hip ratio; IBW, ideal body weight by the Hamwi equation; AdjWt, adjusted body weight; MAC, midarm circumference; TST, triceps skin-fold thickness; MAMA, midarm muscular area; PBF, percent body fat; FM, fat mass; FMI, fat mass index; FFM, fat-free mass; FFMI, fat-free mass index; TBW, total body water; RMR, resting metabolic rate; RQ, respiratory quotient; VO_2_, volume of O_2_ inspired per minute; VCO_2_, volume of CO_2_ expired per minute.

**Table 3:** Accuracy of the resting metabolic rate measured by indirect calorimetry and that calculated by some predictive equations.

Tools	RMR (kcal/day)	Bias (kcal)	Percent of bias (%)	Underestimation (%)	Overestimation (%)	Accurate estimation (%)	*r*
*Men (n* = *208)*							
Quark RMR	1999.1 ± 360.1	—	—	—	—	—	—
HB^1919^	1922.3 ± 288.5^*∗*^	76.8 ± 251.2	4.3 ± 13.4	26.9	17.8	55.3	0.721
HB^1984^	1906.2 ± 280.0^*∗*^	92.8 ± 249.9	5.3 ± 13.4	30.3	14.9	54.8	0.722
Bernstein	1508.1 ± 246.8^*∗*^	491.0 ± 250.0	33.5 ± 17.6	90.4	0	9.6	0.720
Owen	1747.6 ± 204.9^*∗*^	251.5 ± 257.5	14.3 ± 14.6	59.1	3.8	37.0	0.714
Mifflin	1799.6 ± 215.5^*∗*^	199.5 ± 252.8	11.0 ± 14.0	50.5	5.3	44.2	0.723
Schofield (Wt)	1973.6 ± 302.1	24.7 ± 256.3	1.9 ± 13.3	21.6	22.6	55.8	0.714
Schofield (Wt, Ht)	1970.9 ± 302.3	28.2 ± 256.3	1.8 ± 13.3	21.1	21.6	56.3	0.714
WHO (Wt)	1981.9 ± 307.4	17.18 ± 257.2	1.3 ± 13.2	20.2	23.6	56.3	0.714
WHO (Wt, Ht)	1982.3 ± 309.0	16.8 ± 257.7	1.3 ± 13.3	20.7	23.1	56.3	0.713

*Women (n* = *215)*							
Quark RMR	1508.6 ± 241.3	—	—	—	—	—	—
HB^1919^	1470.9 ± 134.3^*∗*^	37.7 ± 178.1	2.4 ± 11.9	20.5	18.1	61.4	0.687
HB^1984^	1479.5 ± 132.9^*∗*^	29.2 ± 178.5	1.8 ± 11.8	19.5	18.1	62.3	0.687
Bernstein	1207.3 ± 102.1^*∗*^	301.3 ± 186.9	24.7 ± 14.7	82.3	0.5	17.2	0.684
Owen	1296.8 ± 103.7^*∗*^	211.9 ± 189.2	16.1 ± 14.0	60.9	1.4	37.7	0.663
Mifflin	1372.9 ± 150.8^*∗*^	135.7 ± 177.4	9.9 ± 12.7	41.9	5.6	52.6	0.680
Schofield (Wt)	1522.2 ± 214.0	−13.6 ± 188.5	−0.4 ± 12.3	17.2	27.0	55.8	0.663
Schofield (Wt, Ht)	1484.2 ± 201.7	24.5 ± 184.5	2.1 ± 12.4	22.8	18.6	58.6	0.666
WHO (Wt)	1523.3 ± 212.3	−14.7 ± 188.0	−0.5 ± 12.3	17.2	27.4	55.3	0.663
WHO (Wt, Ht)	1492.6 ± 198.3	16.0 ± 183.8	1.4 ± 12.3	20.5	20.9	58.6	0.666

Bias = RMR measured by Quark RMR-REE predicted by equations. Percent of bias = ((RMR measured by Quark RMR-REE predicted by equations) × 100)/REE predicted by equations. Accurate estimation = percentage of all subjects whose REE was within 90% to 110% of measured RMR by Quark RMR. Underestimation = percentage of all subjects whose REE was less than 90% of measured RMR by Quark RMR. Overestimation = percentage of all subjects whose REE was more than 110% of measured RMR by Quark RMR. *r* = Pearson's correlation coefficient between RMR measured by Quark RMR and REE predicted by equations. ^*∗*^significant difference vs the measured RMR (Quark RMR) (*P* ≤ 0.05).

**Table 4:** Pearson correlation coefficients for resting metabolic rate (RMR) and other predictive variables.

Independent variables	Total (*n* = 423)	Men				Women			
		Total men (*N* = 208)	Normal Wt (*N* = 59)	Over Wt (*N* = 80)	Obesity (*N* = 69)	Total women (*N* = 215)	Normal Wt (*N* = 75)	Over Wt (*N* = 76)	Obesity (*N* = 64)
Age	−0.230^*∗∗*^	−0.039	0.038	−0.072	−0.141	0.053	−0.120	−0.142	−0.055
Ht	0.614^*∗∗*^	0.299^*∗∗*^	0.477^*∗∗*^	0.238^*∗*^	0.335^*∗∗*^	0.263^*∗∗*^	0.418^*∗∗*^	0.405^*∗∗*^	0.173
Wt	0.750^*∗∗*^	0.714^*∗∗*^	0.352^*∗∗*^	0.378^*∗∗*^	0.659^*∗∗*^	0.663^*∗∗*^	0.348^*∗∗*^	0.465^*∗∗*^	0.694^*∗∗*^
BMI	0.553^*∗∗*^	0.647^*∗∗*^	0.026	0.290^*∗∗*^	0.487^*∗∗*^	0.603^*∗∗*^	0.085	0.272^*∗*^	0.686^*∗∗*^
IBW	0.633^*∗∗*^	0.299^*∗∗*^	0.477^*∗∗*^	0.237^*∗*^	0.335^*∗∗*^	0.263^*∗∗*^	0.418^*∗∗*^	0.405^*∗∗*^	0.173
AdjWt	0.787^*∗∗*^	0.643^*∗∗*^	0.485^*∗∗*^	0.288^*∗∗*^	0.579^*∗∗*^	0.571^*∗∗*^	0.426^*∗∗*^	0.441^*∗∗*^	0.469^*∗∗*^
WC	0.667^*∗∗*^	0.642^*∗∗*^	0.144	0.219	0.535^*∗∗*^	0.538^*∗∗*^	0.037	0.240^*∗*^	0.531^*∗∗*^
HC	0.508^*∗∗*^	0.629^*∗∗*^	0.347^*∗∗*^	0.209	0.585^*∗∗*^	0.556^*∗∗*^	0.284^*∗*^	0.149	0.520^*∗∗*^
WHR	0.394^*∗∗*^	0.155^*∗*^	−0.094	−0.105	−0.007	0.112	−0.244^*∗*^	0.155	0.122
MAC	0.314^*∗∗*^	0.603^*∗∗*^	0.193	0.351^*∗∗*^	0.414^*∗∗*^	0.502^*∗∗*^	−0.028	0.221	0.407^*∗∗*^^*∗∗*^
TST	0.243^*∗∗*^	0.486^*∗∗*^	−0.036	0.081	0.298^*∗*^	0.325^*∗∗*^	−0.233^*∗*^	0.081	0.249^*∗*^
MAMA	0.208^*∗∗*^	0.358^*∗∗*^	0.155	0.271^*∗*^	0.312^*∗∗*^	0.397^*∗∗*^	0.138	0.150	0.267^*∗*^
PBF	−0.038	0.521^*∗∗*^	−0.014	−0.061	0.298^*∗*^	0.458^*∗∗*^	0.201	0.100	0.197
FM	0.415^*∗∗*^	0.641^*∗∗*^	0.107	0.089	0.524^*∗∗*^	0.629^*∗∗*^	0.280^*∗*^	0.336^*∗∗*^	0.661^*∗∗*^
FMI	0.264^*∗∗*^	0.603^*∗∗*^	−0.001	0.019	0.427^*∗∗*^	0.593^*∗∗*^	0.172	0.195	0.642^*∗∗*^
FFM	0.819^*∗∗*^	0.690^*∗∗*^	0.369^*∗∗*^	0.409^*∗∗*^	0.567^*∗∗*^	0.646^*∗∗*^	0.309^*∗∗*^	0.480^*∗∗*^	0.614^*∗∗*^
FFMI	0.746^*∗∗*^	0.614^*∗∗*^	0.042	0.364^*∗∗*^	0.380^*∗∗*^	0.543^*∗∗*^	−0.108	0.205	0.583^*∗∗*^
TBW	0.245^*∗∗*^	−0.288^*∗∗*^	−0.189	−0.071	0.063	−0.225^*∗∗*^	−0.330^*∗∗*^	−0.265^*∗*^	−0.099
VO_2_	0.945^*∗∗*^	0.992^*∗∗*^	0.986^*∗∗*^	0.991^*∗∗*^	0.989^*∗∗*^	0.977^*∗∗*^	0.988^*∗∗*^	0.939^*∗∗*^	0.993^*∗∗*^
VCO_2_	0.991^*∗∗*^	0.914^*∗∗*^	0.810^*∗∗*^	0.901^*∗∗*^	0.872^*∗∗*^	0.899^*∗∗*^	0.819^*∗∗*^	0.903^*∗∗*^	0.878^*∗∗*^

Ht, height; Wt, weight; BMI, body mass index; IBW, ideal body weight by the Hamwi equation; AdjWt, adjusted body weight; WC, waist circumference; HC, hip circumference; WHR, waist hip ratio; MAC, midarm circumference; TST, triceps skinfold thickness; MAMA, midarm muscular area; PBF, percent body fat; FM, fat mass; FMI, fat mass index; FFM, fat-free mass; FFMI, fat-free mass index; TBW, total body water; RMR, resting metabolic rate; VO_2_, volume of O_2_ inspired per minute; VCO_2_, volume of CO_2_ expired per minute; ^*∗∗*^correlation is significant at the 0.01 level (2-tailed); ^*∗*^correlation is significant at the 0.05 level (two-tailed).

**Table 5:** New predictive equations among total population.

Independent variables	Stepwise entry of variables	*R*	*R* square	Adjusted *R* square	Equation
*Total (n* = *423)*					
Age, gender, Ht, Wt, BMI, IBW, AdjWt, WC, HC, WHR, MAC, TST, MAMA	AdjWt	0.787	0.620	0.619	REE = 3832.955 + AdjWt × 48.037 − Ht (cm) × 30.642 + gender^*∗*^ × 141.268 − age × 4.525 (equation AA_1)
	Ht	0.821	0.674	0.672	
	Gender	0.834	0.695	0.693	
	Age	0.838	0.702	0.699	

Gender, PBF, FM, FMI, FFM, FFMI, TBW	FFM	0.819	0.671	0.670	REE = 683.588 + FFM × 21.168 − TBW × 6.119 + gender^*∗*^ × 208.529 + FM × 5.704 (equation AA_FFM)
	TBW	0.832	0.692	0.691	
	Gender	0.835	0.698	0.696	
	FM	0.842	0.708	0.706	

*Normal weight population (n* = *134)*					
Age, gender, Ht, Wt, BMI, WC, HC, WHR, MAC, TST, MAMA	Ht	0.746	0.556	0.553	REE = Ht × 14.923 + gender^*∗*^ × 202.170 − 990.796 (equation AA_2)
	Gender	0.784	0.615	0.609	

Age, gender, PBF, FM, FMI, FFM, TBW	FFM	0.745	0.555	0.551	REE = 1053.737 + FFM × 19.536 − TBW × 6.664 + gender^*∗*^ × 196.446 − age × 6.445
	TBW	0.763	0.582	0.576	
	Gender	0.789	0.622	0.613	
	Age	0.798	0.637	0.625	

*Over Wt and obese (n* = *289)*					
Age, gender, Ht, Wt, BMI, IBW, AdjWt, WC, HC, WHR, MAC, TST, MAMA	AdjWt	0.763	0.582	0.580	REE = 4128.975 + AdjWt × 49.455 − Ht (cm) × 33.117 + gender^*∗*^ × 160.727 − age × 4.207 (equation AA_3)
	Ht	0.798	0.637	0.634	
	Gender	0.813	0.661	0.657	
	Age	0.817	0.668	0.663	

Age, gender, PBF, FM, FMI, FFM, FFMI, TBW	FFM	0.800	0.640	0.639	REE = 712.703 + FFM × 30.735 − TBW × 9.738 − age × 4.180
	TBW	0.815	0.664	0.661	
	Age	0.819	0.671	0.667	

^*∗*^Gender for men use 1 and for women use 0. AdjWt is Adjusted body weight = IBW + 0.25 × (Wt – IBW). IBW is Ideal body weight; for men IBW = (Ht (cm) − 152.4) × 1.0714) + 45.36 and for women IBW = (Ht (cm) − 152.4) × 0.8928) + 45.36.

**Table 6:** New predictive equations among men.

Independent variables	Stepwise entry of variables	*R*	*R* square	Adjusted *R* square	Equation
*All men (n* = *208)*					
Age, Ht, Wt, BMI, IBW, AdjWt, WC, HC, WHR, MAC, TST, MAMA	Wt	0.724	0.524	0.521	REE = 910.062 + Wt × 20.046 − BMI × 21.003
	BMI	0.733	0.537	0.533	
Age, PBF, FM, FMI, FFM, FFMI, TBW	FFM	0.696	0.484	0.482	REE = 591.198 + FFM × 19.555 − FM × 9.219
	FM	0.731	0.534	0.529	

*Normal weight men (n* = *59)*					
Age, Ht, Wt, BMI, WC, HC, WHR, MAC, TST, MAMA	Ht	0.477	0.227	0.214	REE = Ht × 18.035 − 1320.610
Age, PBF, FM, FMI, FFM, TBW	FFM	0.369	0.136	0.121	REE = 1193.657 + FFM × 18.076 − TBW × 6.971
	TBW	0.451	0.204	0.175	

*Over Wt and obese men (n* *=* *149)*					
Age, Ht, Wt, BMI, IBW, AdjWt, WC, HC, WHR, MAC, TST, MAMA	Wt	0.673	0.453	0.449	REE = 852.567 + Wt × 13.342
Age, PBF, FM, FMI, FFM, FFMI, TBW	FFM	0.644	0.415	0.411	REE = 567.886 + FFM × 19.778 + FM × 8.984
	FM	0.682	0.465	0.458	

**Table 7:** New predictive equations among women.

Independent variables	Stepwise entry of variables	*R*	*R* Square	Adjusted *R* square	Equation
*All women (n* = *215)*					
Age, Ht, Wt, BMI, IBW, AdjWt, WC, HC, WHR, MAC, TST, MAMA	Wt	0.663	0.440	0.437	REE = 972.009 + Wt × 15.278 − age × 5.369 − MAC × 11.674
	Age	0.686	0.470	0.465	
	MAC	0.695	0.483	0.476	
Age, PBF, FM, FMI, FFM, FFMI, TBW	FFM	0.646	0.417	0.415	REE = 745.607 + FFM × 24.818 − TBW × 7.185 − age × 5.793 + FMI × 14.472
	TBW	0.705	0.496	0.492	
	Age	0.718	0.516	0.509	
	FMI	0.733	0.537	0.528	

*Normal weight women (n* = *75)*					
Age, Ht, Wt, BMI, WC, HC, WHR, MAC, TST, MAMA	Ht	0.418	0.175	0.164	REE = Ht × 12.086 − 542.676
Age, PBF, FM, FMI, FFM, FFMI, TBW	TBW	0.330	0.109	0.097	REE = 966.832 − TBW × 6.029 + FFM × 22.696 − age × 8.674
	FFM	0.485	0.236	0.214	
	Age	0.559	0.312	0.283	

*Over Wt and obese women (n* = *140)*					
Age, Ht, Wt, BMI, IBW, AdjWt, WC, HC, WHR, MAC, TST, MAMA	Wt	0.602	0.362	0.358	REE = 794.871 + Wt × 12.565 − age × 5.499
	Age	0.628	0.394	0.386	
Age, PBF, FM, FMI, FFM, FFMI, TBW	FFM	0.590	0.348	0.343	REE = 602.841 + FFM × 32.399 − TBW × 12.578
	TBW	0.670	0.449	0.441	

**Table 8:** General characteristics of subjects used for external validation.

Variables	Total	Men	Women	*P* value
	Mean ± SD (*n* = 48)	Mean ± SD (*n* = 24)	Mean ± SD (*n* = 24)	
Age (year)	29.94 ± 8.39	28.42 ± 10.00	31.46 ± 6.25	NS
Ht (cm)	164.33 ± 7.90	170.46 ± 4.85	158.19 ± 5.06	*P* < 0.001
Wt (kg)	79.69 ± 17.40	85.27 ± 19.90	74.11 ± 12.54	*P* < 0.05
BMI (kg/m^2^)	29.44 ± 5.67	29.33 ± 6.83	29.56 ± 4.37	NS
IBW	57.74 ± 8.65	64.84 ± 5.20	50.64 ± 4.52	*P* < 0.001
AdjWt	63.23 ± 9.23	69.95 ± 6.93	56.51 ± 5.62	*P* < 0.001
WC (cm)	89.89 ± 12.95	92.75 ± 16.01	87.03 ± 8.32	NS
HC (cm)	106.12 ± 11.85	104.13 ± 13.89	108.11 ± 9.25	NS
WHR	0.85 ± 0.07	0.89 ± 0.06	0.81 ± 0.06	*P* < 0.001
MAC (cm)	30.61 ± 4.62	29.02 ± 4.83	32.21 ± 3.88	*P* < 0.05
TST (mm)	27.81 ± 8.94	24.56 ± 9.05	31.05 ± 7.72	*P* < 0.05
MAMA (cm^2^)	39.14 ± 13.61	37.20 ± 14.08	41.07 ± 13.14	NS
PBF (%)	32.10 ± 9.52	25.43 ± 8.17	38.78 ± 5.04	*P* < 0.001
FM (kg)	26.06 ± 11.12	22.86 ± 12.62	29.25 ± 8.48	*P* < 0.05
FFM (kg)	53.38 ± 11.08	61.91 ± 8.85	44.85 ± 4.55	*P* < 0.001
TBW (L)	48.30 ± 9.27	55.12 ± 5.42	41.49 ± 7.00	*P* < 0.001
RMR (kcal/day)	1737.52 ± 378.49	1971.17 ± 386.37	1503.86 ± 171.84	*P* < 0.001
RQ	0.74 ± 0.07	0.78 ± 0.06	0.69 ± 0.06	*P* < 0.001

**Table 9:** Accuracy of the new equations vs resting metabolic rate measured by indirect calorimetry and that calculated by some predictive equations in population used for external validation (*n* = 48; men: 50%).

Tools	RMR (kcal/day)	Bias (kcal)	Percent of bias (%)	Underestimation (%)	Overestimation (%)	Accurate estimation (%)	*r*
Measured RMR	1737.52 ± 378.49	—	—	—	—	—	—
AA_1	1770.15 ± 296.80	32.63 ± 219.84	2.00 ± 11.72	14.58	25.00	60.42	*0.815* ^*∗∗*^
AA_FFM	1770.94 ± 313.25	33.41 ± 219.60	1.90 ± 11.49	14.58	22.92	62.50	*0.815* ^*∗∗*^
HB 1919	1704.65 ± 292.18	32.87 ± 227.50	1.81 ± 12.62	16.67	22.92	60.42	*0.800* ^*∗∗*^
Bernstein	1362.08 ± 218.08^*∗∗*^	375.44 ± 248.63	27.32 ± 17.12	87.50	0.00	12.50	*0.781* ^*∗∗*^
Owen	1537.92 ± 263.66^*∗∗*^	199.60 ± 224.21	12.86 ± 13.29	58.33	4.17	37.50	*0.814* ^*∗∗*^
Mifflin	1597.84 ± 256.53^*∗∗*^	139.68 ± 229.47	8.51 ± 13.34	35.42	4.17	60.42	*0.805* ^*∗∗*^

AA_1 and AA_FMM are the newly developed equations. Bias = RMR measured by Quark RMR-REE predicted by equations; percent of bias = [(RMR measured by Quark RMR-REE predicted by equations) ∗ 100]/REE predicted by equations; accurate estimation = percentage of all subjects whose REE was within 90% to 110% of measured RMR by Quark RMR; underestimation = percentage of all subjects whose REE was less than 90% of measured RMR by Quark RMR; overestimation = percentage of all subjects whose REE was more than 110% of measured RMR by Quark RMR; *r*, Pearson's correlation coefficient between RMR measured by Quark RMR and REE predicted by equations.^*∗∗*^Significant (*P* < 0.001).

## Data Availability

The original data used to support the findings of this study are available from the corresponding author upon request.

## Supplementary Materials

Supplementary Materials A simplified calculator for the main equations (AA_1 and AA_FFM). Instruction for use: according to the gender, please either insert the age, weight, and height or the bioelectrical impedance data such as FFM, FM, and TBW and then get the RMR in kcal/day.
